# Modeling the patient and health system impacts of alternative xpert® MTB/RIF algorithms for the diagnosis of pulmonary tuberculosis in Addis Ababa, Ethiopia

**DOI:** 10.1186/s12879-017-2417-6

**Published:** 2017-05-02

**Authors:** Abraham Tesfaye, Daniel Fiseha, Dawit Assefa, Eveline Klinkenberg, Silvia Balanco, Ivor Langley

**Affiliations:** 1 Addis Ababa City Government Health Bureau, Addis Ababa, Ethiopia; 2 KNCV Tuberculosis Foundation, Addis Ababa, Ethiopia; 3 Department of Global Health, Academic Medical Center, Amsterdam Institute for Global Health and Development, University of Amsterdam, Amsterdam, the Netherlands; 4 Addis Ababa University, Addis Ababa, Ethiopia; 5 Liverpool School of Tropical Medicine, Pembroke, United Kingdom

**Keywords:** Modeling, Tuberculosis, Ethiopia, Diagnostics, Cost-effectiveness

## Abstract

**Background:**

To reduce global tuberculosis (TB) burden, the active disease must be diagnosed quickly and accurately and patients should be treated and cured. In Ethiopia, TB diagnosis mainly relies on spot-morning-spot (SMS) sputum sample smear analysis using Ziehl-Neelsen staining techniques (ZN). Since 2014 targeted use of xpert has been implemented. New diagnostic techniques have higher sensitivity and are likely to detect more cases if routinely implemented. The objective of our study was to project the effects of alternative diagnostic algorithms on the patient, health system, and costs, and identify cost-effective algorithms that increase TB case detection in Addis Ababa, Ethiopia.

**Methods:**

An observational quantitative modeling framework was applied using the Virtual Implementation approach. The model was designed to represent the operational and epidemiological context of Addis Ababa, the capital city of Ethiopia. We compared eight diagnostic algorithm with ZN microscopy, light emitting diode (LED) fluorescence microscopy and Xpert MTB/RIF. Interventions with an annualized cost per averted *disability adjusted life year* (DALY) of less than the Gross Domestic Product *(*GDP) per capita are considered cost-*effective* interventions.

**Results:**

With a cost lower than the average per-capita GDP (US$690 for Ethiopia) for each averted *disability adjusted life year* (DALY), three of the modeled algorithms are cost-effective. Implementing them would have important patient, health system, and population-level effects in the context of Addis Ababa

❖ The full roll-out of Xpert MTB/RIF as the primary test for all presumptive TB cases would avert 91170 DALYs (95% credible interval [CrI] 54888 – 127448) with an additional health system cost of US$ 11.6 million over the next 10 years. The incremental cost-effectiveness ratio (ICER) is $370 per DALY averted.

❖ Same day LED fluorescence microscopy for all presumptive TB cases combined with Xpert MTB/RIF targeted to HIV-positive and High multidrug resistant (MDR) risk groups would avert 73600 DALYs( 95% CrI 48373 - 99214) with an additional cost of US$5.1 million over the next 10 years. The ICER is $169per DALY averted.

❖ Same-day LED fluorescence microscopy for all presumptive TB cases (and no Xpert MTB/RIF) would avert 43580 DALYs with a reduction cost of US$ 0.2 million over the next 10years. The ICER is $13 per DALY averted.

**Conclusions:**

The full roll-out of Xpert MTB/RIF is predicted to be the best option to substantially reduce the TB burden in Addis Ababa and is considered cost effective. However, the investment cost to implement this is far beyond the budget of the national TB control program. Targeted use of Xpert MTB/RIF for HIV positive and high MDR risk groups with same-day LED fluorescence microscopy for all other presumptive TB cases is an affordable alternative.

**Electronic supplementary material:**

The online version of this article (doi:10.1186/s12879-017-2417-6) contains supplementary material, which is available to authorized users.

## Background

According to the WHO global tuberculosis (TB) report [1], Ethiopia is one of the 30 high TB burden countries with an estimated prevalence of TB of 200 (161–243) per 100, 000 population. Despite all the efforts and commitments exerted and opportunities availed, the Ethiopian national tuberculosis program (NTP) is still facing many challenges which possibly compromise the coverage and quality of TB prevention and control activities: one of the challenges is low TB case detection rate. A major hurdle contributing to the low TB case detection rate is the lack of an accurate diagnostic algorithm. In Ethiopia screening strategies for presumptive tuberculosis cases mainly depends on a combination of symptom screening and microscopy using 3 samples (spot-morning-spot) and for some cases chest X-ray [2]. This is a major shortcoming as it is now well established that these standard diagnostic tools perform sub-optimally [3]. In one study conducted in Addis Ababa’s health facilities, the sensitivity of Ziehl-Neelsen(ZN) microscopy was found to be 29.2% [4]. Other, studies have demonstrated that the diagnostic delay of smear microscopy resulted in a significant loss-to-follow-up of active TB patients after initial screening [3, 5]. Screening strategies based on culture are more accurate, but are often very expensive, not routinely available and take several weeks to come to diagnosis [2], which compromises patient follow-up and traceability.

Ethiopia is implementing targeted use of Xpert MTB/RIF since 2014 [6], and the utilization is suboptimal. New diagnostic tools for TB have the potential to deliver substantial reductions in the burden of disease through improved sensitivity [7], lower diagnostic default, less delay to starting appropriate treatment and reductions in TB incidence [5]. Thus, the need for full implementation of newer diagnostic approaches for case detection in Ethiopian context is urgent. However, these new tools are more costly than the conventional diagnostic tools. Accordingly, policymakers must decide on the best combination of tools to choose; who should be tested with the new tools; whether the new tools complement or replace the existing diagnostics; and on the general impact of implementing the new tools on patients, health systems and epidemiological burden [8–10]. It would be expensive, time-consuming and disruptive to the health system to try and answer all these questions through experimentation in the live environment. Mathematical modeling has the potential of addressing the above questions to help guide policy makers in the most optimal and cost-effective use of new diagnostic tools [11, 12]. Modeling can project the effect of rolling out the new algorithm on the number of patients diagnosed, treated and cured. This enables each new diagnostic option to be evaluated and compared in terms of the number of *disability adjusted life years* (DALY’s) averted, incremental cost-effectiveness ratio’s (ICER), and sustainability. Different combinations or packages of tests and approaches can be modeled. This study is proposed to assess the impact of alternative diagnostic algorithms on tuberculosis detection using a modeling approach in a district of Addis Ababa, Ethiopia. The analysis adds to other work on this topic [5, 9, 12–14].

## Methods

### Study setting and algorithms

**Fig. 1:**
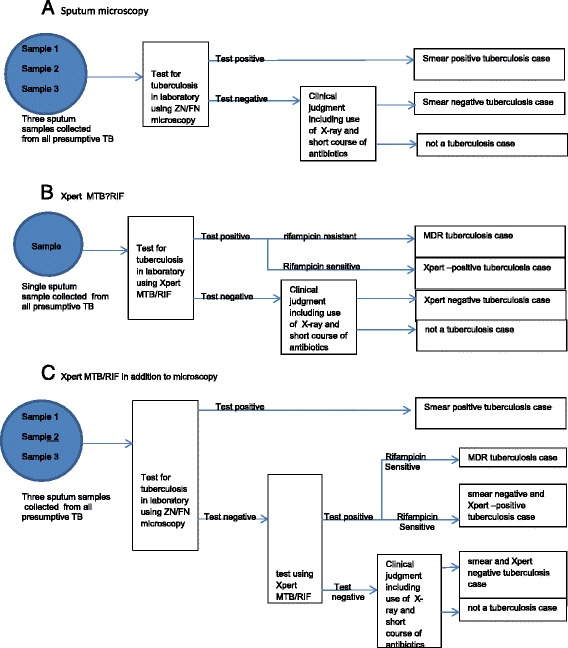
Patient pathway for diagnostic algorithms. **a** Sputum microscopy. **b** Xpert MTB/RIF. **c** Xpert MTB/RIF in addition to microscopy

The study was conducted for public health facilities which provide the Directly Observed Treatment (DOT) services to TB patients in Addis Ababa , Ethiopia. We compared the impact of eight diagnostic algorithms. These were chosen based on WHO [15] and Ethiopian ministry of Health [6] recommendations. Routine diagnostic algorithms, that are common in the country, were also considered. Under the **ZN-Spot-Morning-Spot** scenario, Ziehl-Neelsen (ZN) microscopy using three sputum samples provided within two days was modeled. This scenario represents the common diagnostic algorithms for presumptive TB patients when there is no access to Xpert MTB/RIF (expert opinion). In this model, it was considered as the base case for comparison. **FN-Spot-Morning-Spot** scenario replaces ZN microscopy with light-emitting diode (LED) fluorescence microscopy. **FN-Spot-Morning** and **FN-Spot-Spot** scenarios use LED Fluorescence microscopy using two sputum samples provided within two days (on the SPOT-early MORNING) and on the same day (on the SPOT-SPOT), respectively. **Full-Xpert** scenario tests all patients with presumptive tuberculosis with Xpert. **Targeted-Xpert- MDR-HIV-ZN-Spot-Morning-Spot** scenario uses ZN microscopy (SPOT-MORNING-SPOT) as the primary test for tuberculosis diagnosis and target Xpert for presumptive TB cases that are HIV positive, known contact of MDR-TB patients, and retreatment cases. But **Targeted-Xpert-ZN-Negative-Spot-Morning-Spot** scenario targets the use of Xpert to a patient with presumptive TB who are smear negative in addition to a patient who is HIV positive, known contact of MDR-TB patients, and retreatment case. **Targeted-Xpert-MDR-HIV-FN-Spot-Spot** scenario uses LED fluorescence microscopy on the same day (SPOT-SPOT) as the primary test for tuberculosis diagnosis and target Xpert for presumptive TB cases who are HIV positive, known contact of MDR-TB patients, and retreatment cases. Figure 1 Illustrate the diagnostic algorithms used in the analysis.

### Model structure

We adopted a modeling framework pioneered by Langley et al. in Tanzania [5] to represent the operational and epidemiological context of Addis Ababa, Ethiopia. The model used the discrete-event simulation (DES) approach and Witness software to represent the patient pathways that individual patients follow and the sputum sample pathways in the laboratory.

The patient pathways, the patient was categorized as either new cases or retreatment cases, and HIV+ or HIV- with some knowing their status and some not. Multiple visits to the diagnostic center are required by each new patient. The number of visits for each patient is dependent on a number of factors which were modeled. These factors included the diagnostic test; whether the patient became diagnostic lost to follow-up (LTFU); the backlog in the laboratory that could lead to diagnosis not being available; the time the patient was willing to wait in the diagnostic centre for results; whether the initial test result was positive; whether X-ray and a short course of antibiotics was required following a negative sputum test; and whether TB treatment would be required. Once TB has been diagnosed a further visit to the diagnostic center is required to receive the first batch of medication and for treatment education. Patients return to the diagnostic center during treatment for sputum smear microscopy testing at the end of each phase of treatment. Patients are finally categorized as cured, completed, failed treatment, or treatment lost to follow-up.

In sputum sample pathways, sputum samples are collected in the diagnostic center from presumptive TB cases and individuals undergoing treatment for TB. Samples follow a pathway through the laboratory depending on the patient characteristics, diagnostic technique, and the diagnostic algorithm being used. There were two different types of diagnostic test that were modeled – microscopy and Xpert. These were modeled separately with control logic defining which test process would be used for which patient sample based on the ‘attributes’ of the patient. This logic enabled a number of different diagnostic algorithms to be evaluated. Samples are prepared for the appropriate test, the test is then conducted and a test result assigned.

For simplicity, we assumed a constant population with no immigration or emigration and constant transmission dynamics. Further details on the model and parameter descriptions are given in the Additional file 1 and other references [5, 11]. The model was calibrated by using real data from diagnostic centers in Addis Ababa. The outputs from the model were validated against the observed results from diagnostic centers.

Considering the natural history and pathogenesis of tuberculosis that take generations for the effects of intervention to be seen and situational changes due to new diagnostics technologist in pipeline, we choose a timeframe of 10 years for prediction.

### Diagnostic and cost parameters

A survey of one central referral laboratory and 20 public health facilities that provide TB services provided (randomly selected) estimates for the diagnostic and cost parameters to be used in the model (Additional file 1: Table B). Sensitivity and specificity of diagnostic tools were collated from the literature [16–18] (Additional file 1: Table A). Data were collected from August to December 2014. Additional file 1: part A and B lists the model variables and their sources.

Cost variables and source of the data are presented in Additional file 1: part C. Unit costs were input in US dollars and are based on published data and estimates from Ethiopian National Tuberculosis program, Addis Ababa Health Bureau, and Health facilities. We used public sector salary scale of 2014 to estimate annual employment cost using National Bank of Ethiopia exchange rate (19.7 Birr= 1 USD). Consolidated Health Economic Evaluation Reporting Standards (CHEERS) [19] was used for reporting of economic evaluation results.

### Uncertainty analysis

We conducted uncertainty analysis to estimate the 95% credible interval of projected outcome, accounting for the uncertainty of input parameter values. In the operational component, each simulated year involved running the model for over 2,500 simulated days. Outcomes were recorded for each simulated 90 day period and the mean and standard deviation calculated. From these, 95% confidence limits for key outcome measures were calculated.

### Cost-effectiveness analysis

Cost-effectiveness measures were calculated to compare different diagnostic options. The incremental cost of implementing each alternative diagnostic option was derived from the Addis Ababa health system perspective, and included the additional annual running costs (eg, consumables, susceptible and drug resistant -tuberculosis drugs, radiographs, equipment maintenance, and laboratory personnel) and the investment costs (eg, microscopes and the equipment related to Xpert implementation) (Additional file 1). Other overhead costs were assumed to be unaffected by a change in the diagnostic algorithm. The introduction of new tuberculosis diagnostics is expected to improve the survival of patients co-infected with tuberculosis and HIV, therefore we estimated the incremental costs from additional antiretroviral therapy (ART) on the basis of the projected increase in HIV prevalence resulting from reductions in the number of deaths from tuberculosis with HIV co-infection. The population effect on tuberculosis epidemiology was summarized using *disability- adjusted life years* (DALYs). Costs and DALYs were calculated over 10 years with an annual discount rate of 3%. We calculated the average cost-effectiveness ratio (ACER) by comparing each alternative diagnostic option to the base case scenario. Because the alternative diagnostic options are mutually exclusive interventions that compete for the same resources, we also calculated the incremental cost- effectiveness ratios (ICER) to compare one option with the next less-effective option. The estimated ICER was compared with the willingness-to-pay threshold for Ethiopia (US$690 based on the gross domestic product [GDP] per capita in 2015) [20]. Interventions with an ICER of less than the Gross Domestic Product (GDP) per capita considered as cost-effective interventions [21] but need to be considered alongside affordability, budget impact, fairness, feasibility and any other criteria considred important in the local.

## Results

### Patient-level Impact

The full roll-out of Xpert MTB/RIF is projected to produce the greatest patient-level benefits among the eight alternative diagnostic options (Table 1). The modeling result showed that mean patient visits for diagnosis reduces by an average of 1·4 visits, time to start treatment reduces by an average of 7.8 days, and the diagnostic lost to follow-up rate reduces by 9% compared to the base case (**ZN-Spot-Morning-Spot**). The likelihood that a patient with tuberculosis will successfully complete diagnosis and treatment is increased by 8.3%. The next best algorithms from the patient perspective involve targeting of Xpert MTB/RIF to HIV-positive cases and high MDR risk groups alongside same day LED for the other TB presumptive cases (**Targeted-Xpert-MDR-HIV-FN-Spot-Spot**) and Xpert as a secondary test for smear negative new TB suspects (**Targeted-Xpert-ZN-Negative-Spot-Morning-Spot**).

**Table 1:** Patient-level effects of new diagnostic algorithms

Diagnostic algorithms	Diagnosis and treatment initiation			Diagnosis and treatment success	
	Number of visits to the diagnostic center	Time to start treatment (Days)	Diagnostic LTFU^a^ rate (%)	New tuberculosis cases diagnosed without a positive sputum test (%)	Likelihood of a true tuberculosis patient completing diagnosis and treatment (%)
ZN-Spot-Morning-Spot	5	16.8	13.3	59.8	76.6
FN-Spot-Morning-Spot	4.9	15	12.6	52.9	78.4
Targeted-Xpert-ZN-Negative-Spot-Morning-Spot	4.4	10.5	11.8	28.6	80.9
Targeted-Xpert- MDR-HIV-ZN-Spot-Morning-Spot	4.7	15.2	12.1	52.5	79.2
Full-Xpert	3.6	9	4.3	28.9	84.9
FN-Spot-Spot	4.4	16	8.6	56.6	80.6
FN-Spot-Morning	4.9	15.5	13.1	52.4	78.2
Targeted-Xpert-MDR-HIV-FN-Spot-Spot	4.1	14.6	6.8	49.4	83.3

^a^LTFU-Lost to follow up

### Health system Impact

Under full scale-up of Xpert, the required number of sputum samples per year is 54,000 compared to 113,000 under the base case. The full roll-out of Xpert requires only 33.3% of the laboratory staff time that is needed for the base case (Table 2). There is no significant change in drug sensitive tuberculosis notification among the alternative diagnosis algorithms. After false-positive cases are excluded, full Xpert scale-up is projected to increase the number of true tuberculosis cures by 346 (Table 2). The use of Xpert would also identify 207 rifampicin-resistant cases during the diagnostic process compared with only 59 MDR tuberculosis cases under the base case. Xpert reduces missed TB cases (patients with TB but not diagnosed or lost to follow-up) by 341 patients compared with the base case.

**Table 2:** Health-system-level effects of new diagnostic algorithms: mean per year in 10 years

Diagnostic algorithms	Sputum samples tested for tuberculosis per year			Lab staff utilization (%)	Patients starting tuberculosis treatment per year including false positive			Missed TB^a^ per year	Complete cures per year excluding false positive
	Microscopy (000s)	Xpert (000s)	X-ray (000s)		Standard regimen	Treatment complete/cure	Treatment failure/MDR TB		
ZN-Spot-Morning-Spot	113	0	12	30	6200	5919	59	963	3154
FN-Spot-Morning-Spot	112	0	12	22	5874	5608	59	893	3228
Targeted-Xpert-ZN-Negative-Spot-Morning-Spot	93	33	6	30	5257	4927	148	785	3332
Targeted-Xpert- MDR-HIV-ZN-Spot-Morning-Spot	98	7	11	28	6165	5831	118	859	3263
Full-Xpert	17	37	6	10	5588	5189	207	622	3500
FN-Spot-Spot	86	0	12	17	6269	5984	59	800	3322
FN-Spot-Morning	82	0	12	16	6200	5919	59	963	3154
Targeted-Xpert-MDR-HIV-FN-Spot-Spot	76	7	12	1	5874	5608	59	893	3228

^a^Missed TB- Patient with TB but not diagnosed or lost to follow up

### Cost-effectiveness analysis

**Fig. 2:**
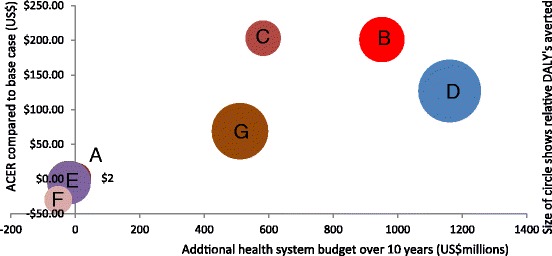
Additional health system budget versus average cost-effectiveness ratio (ACER) versus disability-adjusted life-years (DALYs) averted of alternative diagnostic algorithms. *A*= FN-Spot-Morning-Spot scenario, *B*= Targeted-Xpert-ZN-Negative-Spot-Morning-Spot scenario, *C*= Targeted-Xpert- MDR-HIV-ZN-Spot-Morning-Spot scenario, *D*= Full-Xpert scenario, *E*= FN-Spot-Spot scenario, *F*= FN-Spot-Morning scenario, and *G*= Targeted-Xpert-MDR-HIV-FN-Spot-Spot

The modeling analysis indicated that full roll-out of Xpert is expected to avert approximately 9117 DALYs per years at average cost-effectiveness ratio (ACER) of $127 per *disabilityadjusted life year* (DALY) averted compared to the base case (Table 3). The average cost-effectiveness ratio (ACER) of other alternative diagnostic options ranged from $2 to $203 per DALY averted compared with the base case. Xpert as the primary test for HIV-positive cases and high MDR risk group alongside LED same day for all others (**Targeted-Xpert-MDR-HIV-FN-Spot-Spot**) is expected to avert 7360 DALYs per year and Xpert as secondary tests for smear negative new presumptive TB cases (**Targeted-Xpert-ZN-Negative-Spot-Morning-Spot**) is expected to avert 4724 DALYs. Other diagnostic algorithms would have less effect on DALYs, with the benefits ranging from 1747 (**FN-Spot-Morning**) to 4358 (**FN-Spot-Spot**) DALYs averted per year. The full rollout of Xpert requires an additional budget of an average US$ 1,161,133 budget per year over 10 years. Incremental cost for the other alternative algorithms varies between –US$ 52,727 and US$950,669 over the next 10 years (Table 3, Fig. 2)

**Table 3:** Cost-effectiveness analysis of new diagnostic algorithms

Diagnostic algorithms	DALYs averted per year			Additional health system costs per year over 10 years (US$)	Cost-effectiveness ratio (US$ per DALY averted)	
	YLL	YLD	DALY		ACER	ICER
ZN-Spot-Morning-Spot	0	0	0			
FN-Spot-Morning-Spot	1935	25	1965	3456	2	Dominated
Targeted-Xpert-ZN-Negative-Spot-Morning-Spot	4660	64	4724	950669	201	Dominated
Targeted-Xpert- MDR-HIV-ZN-Spot-Morning-Spot	2838	39	2878	582813	203	Dominated
Full-Xpert	8988	128	9117	1161133	127	370.1
FN-Spot-Spot	4299	59	4358	-19748	-5	12.6
FN-Spot-Morning	1728	25	1747	-52727	-30	-30.2
Targeted-Xpert-MDR-HIV-FN-Spot-Spot	7256	104	7360	510831	69	176.7

*YLL* years of life lost, *YLD* years lost due to disability, *DALY* disability adjusted life year. *ACER* average cost-effectiveness ratio, *ICER* incremental cost-effectiveness ratio

.

In the analysis of the incremental cost-effectiveness ratio (ICER) options **FN-Spot-Morning-Spot**, **Targeted-Xpert-ZN-Negative-Spot-Morning-Spot,** and **Targeted-Xpert-MDR-HIV-ZN-Spot-Morning-Spot** are strongly dominated by same-day LED fluorescence microscopy (**FN-Spot-Spot**), LED same day with Xpert (**Targeted-Xpert-MDR-HIV-FN-Spot-Spot**) , and Xpert full rollout; because they produce DALY gains at a higher incremental cost . The ICER of Xpert full rollout is $370.1 per DALY averted , followed by LED Same Day + Xpert for HIV positive and high MDR risk group (**Targeted-Xpert-MDR-HIV-FN-Spot-Spot**) ($176.7), same-day LED fluorescence microscopy ($12.6) and LED fluorescence microscopy with two sample (-$30.2) (Table 3). The ICERs for the above alternative algorithms are lower than the annual per-capita GDP of Ethiopia (US$690) [20] and are deemed cost-effective.

## Discussion

In this study, we used a modeling approach to evaluate the potential patient and health system effects and to complete a cost effective analysis of eight diagnostic algorithms. Our results indicated that full roll-out of Xpert in Addis Ababa city would have the greatest patient-level and health system benefits, and would be cost effective with the highest DALYs averted among the alternative diagnostic options. The improved diagnostic sensitivity and the need for only one sputum sample for Xpert reduce mean patient visits for diagnosis, time to start treatment, and diagnostic lost to follow-up rate. This result in an increase in the likelihood that a patient with TB will successfully complete diagnosis and treatment. It would result in an additional health system cost, and an incremental cost to the HIV program due to longer life expectancy for co-infected TB and HIV patients. We note that cost-effectiveness does not necessarily mean that the implementation is affordable for the Addis Ababa tuberculosis control program.

A more affordable cost effective alternative is targeted use of Xpert MTB/RIF for HIV positive and high MDR risk groups with same-day LED fluorescence microscopy for all other presumptive tuberculosis cases (**Targeted-Xpert-MDR-HIV-FN-Spot-Spot**). The result is in line with the current effort of national tuberculosis program to scale-up LED fluorescent microscopy and targeted use of Xpert. The third cost effective alternative is same day LED fluorescence microscopy (**FN-Spot-Spot**). The improved diagnostic sensitivity of LED microscopy, and the need for sputum with in the same day reduce diagnostic lost-to follow up rate. This result in better performance of this option as compared to ZN microscopy with spot-morning, and FN spot-morning. The weak part of this option is do not have a room for drug resistant TB.

The other options are not cost effective, with DALY gains at a higher incremental cost and additional health system costs while they are less effective than the above three for the context of Addis Ababa, Ethiopia. The World Health Organization has endorsed the use of same day LED fluorescent microscopy in 2011 [16]. However, in Ethiopia it has not been implemented. Our result also shows that a diagnosis based on 3 (SMS) samples is not cost effective compared to Spot-spot LED fluorescence microscopy. It requires additional patient visits for diagnosis, sputum, and laboratory staff time. It results in higher diagnostic lost to follow-up rate, higher patient and health system costs, and a decrease in the likelihood that a patient with tuberculosis will successfully complete diagnosis and treatment. The national technical working group should discuss this issue for giving advice to policy makers on the most optimal and cost-effective diagnostic algorithm.

Our results are in line with the findings of Langley et al. in Tanzania [1] where projections due to full Xpert rollout meant patient visits for diagnosis reduced by 1.2 (95% CrI 1.1 – 1.3)visits, time to start treatment by 6·6 (95% CrI 5·9–7·3) days, and the diagnostic lost to follow-up rate by 7% (95% CrI 6–9), the likelihood that a patient with tuberculosis will successfully complete diagnosis and treatment increased by 18%. They also reported highest DALY averted, and a reduction of the number of sputum samples by 34%, and laboratory staff time by 45%. In both studies, the effect of the alternative algorithms on tuberculosis case notification was the same and marginal. The reason behind this is that the increase in case detection is counterbalanced by a reduction in the number of false positives. Our results are also in line with other studies ([22, 23]) where novel diagnostics can substantially reduce TB transmission.

We found a scenario whereby targeted use of Xpert is cost effective (**Targeted-Xpert-MDR-HIV-FN-Spot-Spot**). This is cost effective because besides the targeted use we introduce same day LED fluorescence microscopy to those not benefitting from Xpert. The other algorithms with targeted use of Xpert (**Targeted-Xpert-ZN-Negative-Spot-Morning-Spot** & **Targeted-Xpert-MDR-HIV-ZN-Spot-Morning-Spot**) are not cost effective compared to all other options. The reason for the inferior cost-effectiveness is that the projected costs are substantially higher while the projected gains in DALYs are lower than same day LED fluorescence microscopy in combination with Xpert. Similarly, in the Tanzania study targeting of Xpert either to HIV positive or smear negative HIV-positive cases was not cost-effective [5].

A number of limitations were observed in our modeling analysis. All epidemiological modeling results are uncertain [22]. It is affected by uncertainty of variables and assumptions on model structures. Uncertainty analyses were done only for few parameters. Data accuracy can be a constraint with modeling. Full health system costing was not considered. Users of the modeling result should take account that a modeling analysis cannot consider all the practical challenges that might arise from implementation of a new diagnostic methods.

## Conclusions

In conclusion, we suggest that Ethiopian TB program to consider switching to Same-day diagnosis according to WHO recommendation on using same day LED fluorescent microscopy on two spot samples. The full roll-out of Xpert MTB/RIF was predicted to be the best option to substantially reduce the TB burden in Addis Ababa and was considered cost effective. However, the required resources to implement this are beyond the budget of the TB control program. Targeted use of Xpert MTB/RIF for HIV positive and high MDR risk groups with same-day LED fluorescence microscopy for all other presumptive TB cases is an affordable alternative. In a broader perspective, our results also highlight the importance of a modeling approach to support the selection and implementation of the optimal diagnostic algorithm and outlined the delicate balance between maximizing impact but coming up with an affordable diagnostic algorithm option.

## Additional file

Additional file 1: It is key input variables, element types, and additional parametrs to the model. (DOCX 39 kb)

## Abbreviations

ACER: Average cost-effectiveness ratio
ART: Antiretroviral therapy
CHEERS: Consolidated Health Economic Evaluation Reporting Standards
CrI: Credible interval
DALYs: Disability adjusted life years
DES: Discrete-event simulation
DOT: Directly Observed Treatment
GDP: Gross Domestic Product
ICER: Incremental cost-effectiveness ratio
LED: Light emitting diode
LTFU: Lost to follow-up
MDR: Multidrug resistant
NTP: National tuberculosis program
SMS: Spot-morning-spot
TB: Tuberculosis
TRAC/FMOH: Tuberculosis Research Advisory Committee of Federal Ministry of Health
US: United State
USAID: United States Agency for International Development
USD: United State dollars
WHO: World Health Organization
ZN: Ziehl-Neelsen
