# Supplementary material for: Modeling the patient and health system impacts of alternative xpert® MTB/RIF algorithms for the diagnosis of pulmonary tuberculosis in Addis Ababa, Ethiopia
Source: BMC Infect Dis. 2017 May 2;17:318. doi: 10.1186/s12879-017-2417-6 (PMC5414345; doi:10.1186/s12879-017-2417-6)
Supplement: Additional file 1: — It is key input variables, element types, and additional parametrs to the model. (DOCX 39 kb) [file 12879_2017_2417_MOESM1_ESM.docx]

**Additional file**

**Modeling the Patient and Health System Impacts of Alternative Xpert® MTB/RIF Algorithms for the Diagnosis of Pulmonary Tuberculosis in Addis Ababa, Ethiopia**

Abraham Tesfaye^1^ , Daniel Fiseha^2^ , Dawit Assefa^2^ , Eveline Klinkenberg^2,3^ ,

Silvia Balanco^4^ , Ivor Langley ^5^

1. Addis Ababa City Government Health Bureau, Ethiopia, 2.KNCV Tuberculosis Foundation, Ethiopia, 3. Department of Global Health, Academic Medical Center, Amsterdam Institute for Global Health and Development, University of Amsterdam, the Netherlands, 4. Addis Ababa University, Ethiopia, 5. Liverpool School of Tropical Medicine, United Kingdom.

Contents

[Supplement material 1: Key input variables to the model 2](#_Toc463102355)

[A. Sensitivity and specificity data 2](#_Toc463102356)

[B. Base case data 3](#_Toc463102357)

[C. Costs variables 4](#_Toc463102358)

[Supplement material 2: Element types 5](#_Toc463102359)

[Supplement material 3: Additional parameters 6](#_Toc463102360)

# Additional file 1: Key input variables to the integrated model

## Sensitivity and specificity data

| Component | HIV status | Zn microscopy | LED microscopy | Xpert MTB/RIF | Source |
| --- | --- | --- | --- | --- | --- |
| Sensitivity of test for TB | HIV+ | 44.6% | 50.6% | 80.0% | **[**[**1**](#_ENREF_1)**]** |
|  | HIV- | 72.3% | 78.3% | 89.0% |  |
| Specificity of test for TB | HIV+ | 100% | 100% | 97% |  |
|  | HIV- | 99.4% | 99.4% | 99% |  |
| Sensitivity of clinical diagnosis for test negative suspects | HIV+ | 51.9% | 51.9% | 80% | **[**[**2**](#_ENREF_2)**]** |
|  | HIV- | 51.9% | 51.9% | 80% |  |
| Sensitivity improvement of LED fluorescence microscopy over ZN microscopy |  | +6·0% |  |  | **[**[**3**](#_ENREF_3)**]** |
| Specificity improvement of LED fluorescence microscopy over ZN microscopy |  | 0·0% |  |  | **[**[**3**](#_ENREF_3)**]** |
| Sensitivity improvement of the 3^rd^ sputum over two sputum |  | 0.2% |  |  | **(7)** |

## Base case data

|  | value | Source |
| --- | --- | --- |
| Annual new smear-positive and tuberculosis cases per health center | **46** | **[**[**4**](#_ENREF_4)**], survey** |
| Annual new smear-negative and tuberculosis cases per health center | **67** | **Through survey** |
| Annual number of retreatment patients per health center | **7** | **Through survey** |
| Proportion of new presumptive TB cases that are smear positive at diagnosis | **9.0%** | **Through survey** |
| Proportion of tuberculosis cases needing re-treatment | **2%** | **Through survey** |
| Proportion of HIV-positive tuberculosis cases | **25.6%** | **Through survey** |
| Proportion of smear-negative presumptive tuberculosis cases that had radiograph and antibiotic trial | **50%** | **Through survey** |
| MDR tuberculosis in new tuberculosis cases | **2.7%** | **[**[**5**](#_ENREF_5)**]** |
| MDR tuberculosis in retreated tuberculosis cases | **17%** | **[**[**5**](#_ENREF_5)**]** |
| Diagnostic lost to follow-up rate | **13.5%** | **Through survey** |
|  | | |

## Costs variables

|  | Values | Source |
| --- | --- | --- |
| LED fluorescence microscope | $1250·0 | **[**[**6**](#_ENREF_6)**]** |
| Xpert cartridge cost per test | $9.98 | **[**[**6**](#_ENREF_6)**]** |
| Xpert MTB/RIF machine 4 cell | **$17500** | **[**[**6**](#_ENREF_6)**]** |
| Xpert annual maintenance 4 cell | **$1800** | **[**[**6**](#_ENREF_6)**]** |
| Drug sensitivity cost per test | **$19** | **Ethiopian NTP** |
| Microscopy cost per test | **$1.5** | **Ethiopian NTP** |
| Radiograph | **$6.9** | **Ethiopian NTP** |
| Monthly drug cost for standard regimen | **$3** | **Ethiopian NTP** |
| Monthly drug cost for retreatment regimen | **$18.8** | **Ethiopian NTP** |
| Monthly drug cost for MDR tuberculosis regimen | **$119.4** | **Ethiopian NTP** |
| Annual employment costs for a laboratory technician | **$3200** | **Addis Ababa Health Bureau** |
| Annual employment costs for a laboratory assistant | **$2400** | **Addis Ababa Health Bureau** |

LED-light emitting diode. ZN- Ziehl Neelsen. WHO- World Health organization. MDR- Multi drug resistant NTP- National tuberculosis program

According to Witness DES model, ‘*entities*’ , ‘*attributes*’ , ‘*activities*’, ‘*queues*’ and ‘*resources*’ were defined (supplemental material 2). In addition, the DES Witness model developed in this study contained ‘***variables***’ (where input parameters read in from Excel spreadsheets and outputs were recorded), ‘***histograms***’ (to graphical report the distribution of particular outputs) and ‘***time series***’ (to graphical record outputs over time).

# Additional file 2: Element types

| \| **Element type** \| \| --- \| | **Used to represent** |
| --- | --- | --- |
| Entities: | Patients (Presumptive TB cases and TB cases)  Sputum samples |
| Attributes: | HIV status  TB status  New or retreatment presumptive TB case  Test result  Time diagnosis began |
| Activities: | Patients  - Reception  - Sputum Collection  - Direct Observed Treatment Short Course (DOTS) training  - Receiving diagnosis  - Treatment stages  Sputum samples  - Prepare sample for microscopy  - Stain  - Complete microscopy examination  - Prepare for Xpert  - Load Xpert machine  - Run Xpert machine  - Complete Xpert test |
| Queues: | Patients  - Waiting at reception  - Waiting for sputum collection, diagnosis, or DOTS training  - Waiting before returning from home to provide sample or receive diagnosis  Sputum sample  - Waiting for preparation  - Waiting for staining  - Waiting for examination  - Results waiting to communication to patient |
| Resources | Laboratory technicians  Laboratory technologist |

# Additional file 3: Additional parameters

| \| **Input Parameter** \| \| --- \| | **Description** | **Value** | **Source** |
| --- | --- | --- | --- | --- |
| **Diagnostic Centre**  Working days  Minutes in day  Max wait time | No. of workings days in 1 year  No. of working minutes in day  Maximum time a patient will wait for diagnosis. | 251 days  480 minutes  90 minutes |  |
| **Processing Times**  Diagnostic Centre process times | Duration of processes at the diagnostic centre  - Sample Collection  - Clinician time - 3  - DOTS training – 10 | 10 Minutes  3 Minutes  10 Minutes |  |
| Laboratory process times Microscopy | Batch Stain – ZN  - LED  - Overall drying time  - Examination time  - Smear+ ZN  - Smear+ LED  - Smear- ZN  - Smear - LED | 14 minutes  24 minutes  45 minutes  5 minutes  2.5 minutes  10 minutes  2.5 minutes | 8, 9 |
| Laboratory process times Xpert MTB/RIF | \| Preparation time  - Test time  - Examine time \| \| --- \| | 15 minutes  115 minutes  0.5 minutes | 10 |
| X-ray | Time allowed to get X-ray | 1 day |  |
| **Key Probabilities**  Treatment follow-up test is Smear Positive | Standard Regimen after  - Intensive Phase  - Continuation Phase 1  - Continuation Phase 2  Retreatment Regimen  - Intensive Phase  - Continuation Phase 1  - Continuation Phase 2 | 5%  5%  0.2%  8%  0.2%  0.1% |  |
| HIV | HIV Status unknown at point of TB diagnosis | 89.6% |  |
| X-Ray | Requested following a negative smear  Requested following a negative Xpert | 70%  25% |  |
| GeneXpert | Failure Rate | 2% | 10 |
| **Treatment Times**  Standard Regimen  Retreatment Regimen  MDR-TB Regimen | Intensive Phase  Continuation Phase 1  Continuation Phase 2  Intensive Phase  Continuation Phase 1  Continuation Phase 2  Overall time | 2 Months  3 Months  1 Month  3 Months  2 Months  3 Months  24 Months | 11 |
| Short course antibiotics | Time to complete a short course of antibiotics for smear negative patients with TB symptoms | 2 weeks |  |
| **Laboratory Staffing assumptions**  Availability  Numbers  Xpert staffing | Lab staff will be available for the full working day  Enough staff will be available to avoid bottlenecks of longer than 10 days. If not achieved increased staffing will be used and the model re-run  The same level of staffing will be in place if Xpert is implemented as are available currently. Utilization recorded and cost of staffing will be pro-rated by change in utilization. | 480 minutes per day & 251 days per year.  Variable by diagnostic center.  As above |  |

Reference

1. Boehme, C.C., et al., *Feasibility, diagnostic accuracy, and effectiveness of decentralised use of the Xpert MTB/RIF test for diagnosis of tuberculosis and multidrug resistance: a multicentre implementation study.* Lancet, 2011. **377**(9776): p. 1495-505.

2. Swai, H.F., F.M. Mugusi, and J.K. Mbwambo, *Sputum smear negative pulmonary tuberculosis: sensitivity and specificity of diagnostic algorithm.* BMC Res Notes, 2011. **4**(475): p. 1756-0500.

3. WHO, *Fluorescent light-emitting diode (LED) microscopy for diagnosis of tuberculosis : policy statement.* 2011.

4. Addis Ababa Health Bureau, *Annual report.* 2015.

5. Ethiopian Tuberculosis Program, *Annual report*. 2015.

6. Diagnostics, F.o.I., *Price for Xpert® MTB/RIF and FIND country list*, 2013.

7. [Philip Mathew](https://www.ncbi.nlm.nih.gov/pubmed/?term=Mathew%20P%5BAuthor%5D&cauthor=true&cauthor_uid=12202598), [Yen-Hong Kuo](https://www.ncbi.nlm.nih.gov/pubmed/?term=Kuo%20YH%5BAuthor%5D&cauthor=true&cauthor_uid=12202598), [Bindu Vazirani](https://www.ncbi.nlm.nih.gov/pubmed/?term=Vazirani%20B%5BAuthor%5D&cauthor=true&cauthor_uid=12202598), [Robert H. K. Eng](https://www.ncbi.nlm.nih.gov/pubmed/?term=Eng%20RH%5BAuthor%5D&cauthor=true&cauthor_uid=12202598), and [Melvin P Weinstein](https://www.ncbi.nlm.nih.gov/pubmed/?term=Weinstein%20MP%5BAuthor%5D&cauthor=true&cauthor_uid=12202598), *Are Three Sputum Acid-Fast Bacillus Smears Necessary for Discontinuing Tuberculosis Isolation?* [J Clin Microbiol](https://www.ncbi.nlm.nih.gov/pmc/articles/PMC130719/). 2002 **40**(9): 3482–3484.

8. Ramsay A, Cuevas LE, Mundy CJ, Nathanson CM, Chirambo P, Dacombe R, et al., New policies, new technologies: modeling the potential for improved smear microscopy services in Malawi. PLoS One. 2009. **4**(11):e7760.

9. Welsh Office Cardiff, WELCAN UK workload measurement system for pathology: Manual with schedule of unit values. Cardiff, Great Britain1990.

10. Cepheid Xpert MTB/RIF instruction manual

11. World Health Organization. Treatment of tuberculosis guidelines, Fourth Edition. Geneva: World Health Organization 2010.
